# A novel real time imaging platform to quantify macrophage phagocytosis

**DOI:** 10.1016/j.bcp.2016.07.011

**Published:** 2016-09-15

**Authors:** Theodore S. Kapellos, Lewis Taylor, Heyne Lee, Sally A. Cowley, William S. James, Asif J. Iqbal, David R. Greaves

**Affiliations:** Sir William Dunn School of Pathology, South Parks Road, OX1 3RE Oxford, UK

**Keywords:** BMDM, Bone Marrow-derived Macrophage, cDNA, complementary DNA, Ct, Cycle threshold, DAMP, Danger-associated Molecular Pattern, FBS, Fetal Bovine Serum, GEO, Gene Expression Omnibus, hiPS cell, human induced Pluripotent Stem cell, IFN-γ, Interferon-γ, IgG, Immunoglobulin G, IL, Interleukin, LPS, Lipopolysaccharide, MARCO, Macrophage Receptor with Collagenous structure, PAMP, Pathogen-associated Molecular Pattern, PMA, Phorbol 12-myristate-13-acetate, PRR, Pathogen Recognition Receptor, qPCR, quantitative Polymerase Chain Reaction, TLR, Toll-like Receptor, Macrophage, Phagocytosis, Inflammation, LPS

## Abstract

Phagocytosis of pathogens, apoptotic cells and debris is a key feature of macrophage function in host defense and tissue homeostasis. Quantification of macrophage phagocytosis *in vitro* has traditionally been technically challenging. Here we report the optimization and validation of the IncuCyte ZOOM® real time imaging platform for macrophage phagocytosis based on pHrodo® pathogen bioparticles, which only fluoresce when localized in the acidic environment of the phagolysosome. Image analysis and fluorescence quantification were performed with the automated IncuCyte™ Basic Software. Titration of the bioparticle number showed that the system is more sensitive than a spectrofluorometer, as it can detect phagocytosis when using 20× less *E. coli* bioparticles. We exemplified the power of this real time imaging platform by studying phagocytosis of murine alveolar, bone marrow and peritoneal macrophages. We further demonstrate the ability of this platform to study modulation of the phagocytic process, as pharmacological inhibitors of phagocytosis suppressed bioparticle uptake in a concentration-dependent manner, whereas opsonins augmented phagocytosis. We also investigated the effects of macrophage polarization on *E. coli* phagocytosis. Bone marrow-derived macrophage (BMDM) priming with M2 stimuli, such as IL-4 and IL-10 resulted in higher engulfment of bioparticles in comparison with M1 polarization. Moreover, we demonstrated that tolerization of BMDMs with lipopolysaccharide (LPS) results in impaired *E. coli* bioparticle phagocytosis. This novel real time assay will enable researchers to quantify macrophage phagocytosis with a higher degree of accuracy and sensitivity and will allow investigation of limited populations of primary phagocytes *in vitro*.

## Graphical abstract

**Figure:**
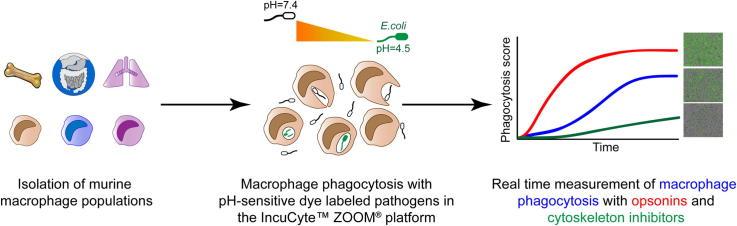

## 1 Introduction

Macrophages are innate immune cells that respond to inflammation, tissue trauma or infection. They were first identified in the nineteenth century by Elie Metchnikoff in sea-star larvae for their ability to surround and engulf a rose thorn, a process he termed phagocytosis [1]. Together with monocytes and dendritic cells, macrophages make up the mononuclear phagocyte network and play an important role in tissue homeostasis [2]. Macrophages are equipped with a range of highly conserved pattern recognition receptors (PRRs) which bind to conserved pathogen-associated molecular patterns (PAMPs), such as lipopolysaccharide (LPS) on Gram-negative bacteria [3], [4]. Similarly, danger-associated molecular patterns (DAMPs), such as high mobility group box 1 [5], IL-1α [6] and uric acid [7] are released by necrotic cells during sterile injury and trigger the activation of macrophages.

Upon PRR engagement, receptor clustering brings together tyrosine kinases which initiate the internalization of foreign particles [8], [9], [10], [11]. Downstream signaling induces the engulfment of the foreign object via protrusions of the cell membrane to form a membrane-bound vesicle [12], [13], [14]. The newly formed vesicle, called the phagosome, undergoes maturation whereby it fuses with several compartments of the endocytic system as it is transported within the cytosol [15]. Earlier work has shown that during maturation, phagosomes acquire numerous proteins, including hydrolases, proton pump ATP subunits and endosome fusion proteins [16]. Changes in the phagosome membrane and its content are continuous, well-orchestrated and eventually lead to its acidification [17], [18].

When phagosome maturation is complete, these organelles fuse with lysosomes which carry an arsenal of hydrolytic enzymes and anti-microbial peptides [19]. The acidic and highly oxidative environment of the phagolysosome is essential for the activation of cathepsins, oxidants and cationic peptides which eventually lead to pathogen lysis and clearance [20].

Researchers have employed a variety of assays to study phagocytosis, including light and confocal microscopy, flow cytometry and imaging flow cytometry and spectrofluorometry. However, technical and practical limitations are associated with all of these techniques, such as dye quenching, low throughput, single end point readouts and often subjective quantification. ESSEN BioScience developed the IncuCyte ZOOM®, a platform which utilizes microscopy to monitor phagocytosis in real time. In this study we report the optimization and validation of this live cell imaging platform to quantify primary murine and human macrophage phagocytosis. We show that this platform is 20 times more sensitive than spectrofluorometry and can detect signals from limited primary macrophage populations.

## 2 Materials and methods

### 2.1 Reagents

Fetal Bovine Serum (FBS), LPS (O111:B4), BSA, RPMI-1640 medium, phorbol 12-myristate-13-acetate (PMA), Bafilomycin A1, Nocodazole and Cytochalasin D were purchased from Sigma-Aldrich (Gillingham, Dorset, UK). PBS was from Lonza (Slough, UK). EDTA was purchased from VWR technologies (East Grinstead, UK). OptiMEM I reduced serum medium, Live Cell Imaging Solution, Penicillin/Streptomycin, green pHrodo® *E. coli* and red *S. aureus* Bioparticles® were from Life Technologies (Paisley, UK). Sterile IncuCyte™ green pHrodo® *E. coli* and Zymosan were purchased from ESSEN Bioscience (Welwyn Garden City, UK). Cytokines (Interferon-γ (IFN-γ), interleukin -4 (IL-4) and IL-10) were from Peprotech (London, UK).

### 2.2 Cell lines

Cell lines were originally from American Type Culture Collection. The RAW264.7 and THP-1 cell lines were a kind gift from Prof. Siamon Gordon, the BV-2 cell line was kindly provided from Prof. David Vaux. Macrophage cell lines at passage number 5 were cultured in RPMI-1640 medium containing 10% FBS and 1% Penicillin/Streptomycin at 37 °C/5% CO_2_. Cells were passaged every three days. THP-1 cells were stimulated with 50 ng/ml PMA for four days before the phagocytosis assay.

### 2.3 Animals

All animal experiments were conducted with local ethical approval from the Dunn School of Pathology Local Ethical Review Committee and in accordance with the UK Home Office regulations (Guidance on the Operation of Animals, Scientific Procedures Act, 1986). Male 8–10 week old C57BL/6J mice (25–30 g) were purchased from Harlan Laboratories (Bicester, UK). All animals were housed in a 12 h light/dark cycle unit with free access to food and water.

### 2.4 Bone marrow-derived macrophages (BMDMs)

BMDMs were generated as previously described [21]. Briefly, tibiae and femurs from male C57BL/6J mice were flushed with PBS and bone marrow cells were re-suspended in Dulbecco Modified Eagle’s Medium supplemented with 10% heat-inactivated FBS, 10–15% L929-conditioned medium [22] and 1% Penicillin-Streptomycin. Cells were cultured for seven days at 37 °C/5% CO_2_ and were re-fed on day 3.

### 2.5 Human induced Pluripotent Stem (hiPS) cell-derived macrophages

The hiPS cell line AH017-13 was derived from dermal fibroblasts of healthy donors recruited by the Oxford Parkinson’s Disease Centre (Ethics committee: National Health Service, Health Research Authority, NRES Committee South Central – Berkshire, UK – REC 10/H0505/71), reprogrammed using standardized protocols in the James Martin Stem Cell Facility, Sir William Dunn School of Pathology, and their SNP datasets and transcriptome array results are deposited in Gene Expression Omnibus (GEO) under accession numbers GSE 53426 [23]. For this study, hiPSCs were thawed and cultured as described in [23]. Differentiation to macrophages via embryoid body formation and directed differentiation was as previously described [24].

### 2.6 IncuCyte ZOOM® phagocytosis assay

**Fig. 1:**
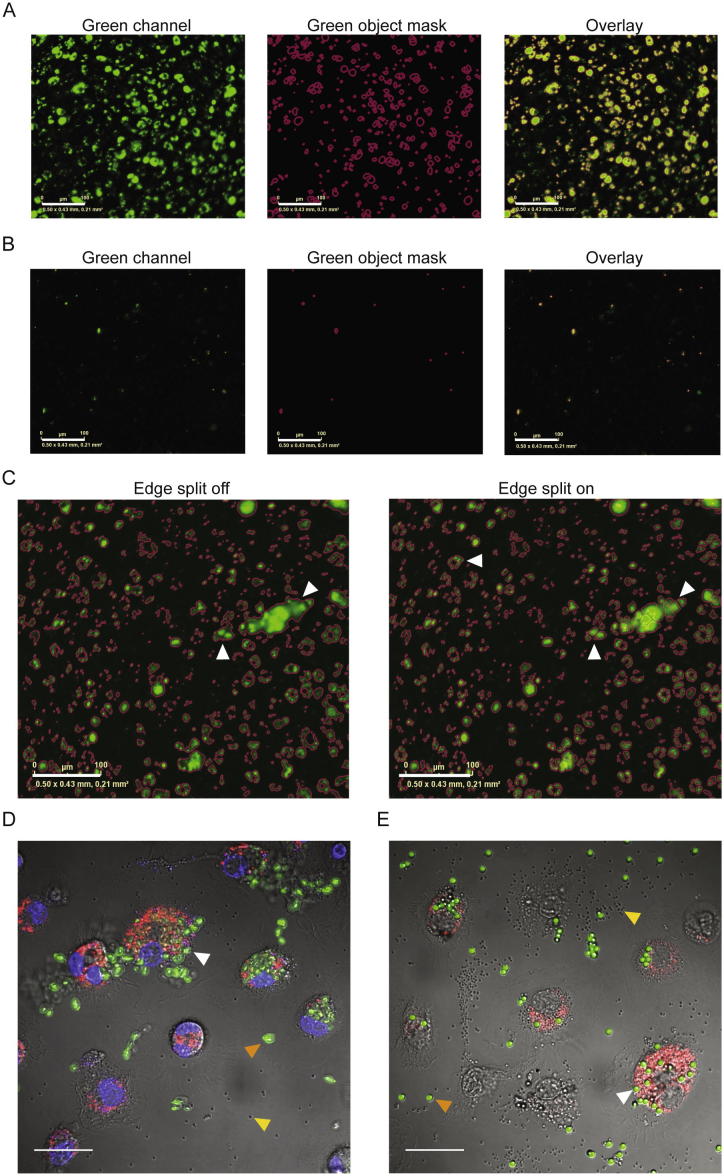
Fluorescent object segmentation analysis by the IncuCyte Basic Software. BMDMs (10^5^) from male C57BL/6J mice were incubated with 1 mg/ml (A) or 10 μg/ml (B) green *E. coli* bioparticles and fluorescence emission was measured in the IncuCyte imaging platform for one hour at 37 °C. Representative images showing the green channel signal (left), the green object mask which was applied for quantification (middle) and the overlay of the two (right). (C) The edge split setting in the IncuCyte Basic Software allows for quantification of closely-spaced objects in both fluorescence channels. Images from 100,000 BMDMs incubated with 1 mg/ml green *E. coli* bioparticles for one hour at 37 °C without (left) or with (right) edge split. The magenta lines delineate separate objects. Scale bar 100 μm. BMDMs (2.5 × 10^4^ per well) were incubated with an equal mix of 200 μg/ml red pHrodo *S. aureus* bioparticles and 200 μg/ml FITC-conjugated Zymosan A (D) or FITC-conjugated 2 μm latex beads (E) and phagocytosis was studied at one hour using confocal microscopy. White arrows indicate FITC-conjugated Zymosan A or latex beads on the macrophage membrane, orange arrows indicate unphagocytosed FITC-conjugated Zymosan A or latex beads and yellow arrows indicate unphagocytosed *S. aureus* bioparticles. NucBlue Live ReadyProbes reagent was used for nuclei staining in (D). Images are from one representative of three independent experiments. Scale bar 20 μm.

Day 7 BMDMs in OptiMEM medium were plated into 96-well flat clear bottom black walled polystyrene tissue-culture treated microplates (Corning, Flintshire, UK) and allowed to adhere for 2 h. pHrodo® pathogen bioparticles were added at indicated concentrations and the plates were transferred into the IncuCyte ZOOM® platform which was housed inside a cell incubator at 37 °C/5% CO_2,_ until the end of the assay. Two images per well from two technical replicates were taken every 10 min for 1 h using a 20× objective lens and then analyzed using the IncuCyte™ Basic Software. Green channel acquisition time was 400 ms, whereas red channel acquisition time was 800 ms. In phase contrast, cell segmentation was achieved by applying a mask in order to exclude cells from background. An area filter was applied to exclude objects below 50 μm^2^. Green and red channel background noise was subtracted with the Top-Hat method of background non-uniformity correction with a radius of 20 μm [25] and a threshold of 2 green and red corrected units. Fluorescence signal was quantified applying a mask (Fig.1A; 1 mg/ml bioparticles, Fig.1B; 10 μg/ml bioparticles). Moreover, as seen in Fig.1C, in the absence of the edge split tool the software recognizes the indicated objects as one, whereas when edge split is applied, the objects are recognized as multiple closely-spaced objects. Edge split was therefore used as a more accurate quantification of fluorescent objects.

### 2.7 Confocal microscopy

BMDMs (2.5 × 10^4^ cells per well) were seeded into IBIDI μ-plate 96 well plates (Munich, Germany) in OptiMEM medium and allowed to adhere for 2 h. Media were removed and replaced with either 200 μg/ml pHrodo red *S. aureus* bioparticles and 200 μg/ml FITC-conjugated Zymosan A or FITC-conjugated 2 μm latex beads (Polysciences Inc, PA, USA) diluted in Live Cell Imaging Solution. Cells were incubated for 1 h at 37 °C 5% CO_2_, followed by extensive washing. NucBlue® Live ReadyProbes® Reagent in live cell imaging solution (ThermoFisher Scientific, MA, USA) was added to stain nuclei. Images were acquired with a 100× objective lens using an Olympus FV1200 confocal microscope (Olympus, PA, USA) fitted with a temperature controlled stage set to 37 °C and analyzed with ImageJ software v1.49.

### 2.8 Spectrofluorometry

Macrophages (1 × 10^5^ cells per well) were plated into a 96-well plate (Corning) and incubated for 1 h with *E. coli* pHrodo bioparticles at 37 °C/5% CO_2_. Excess bioparticles were removed from each well by washing three times with Live Cell Imaging Solution before the measurements were made. Phagocytosis was measured using a PHERAstar spectrofluorometer (BMG LABTECH, Aylesbury, UK) with an excitation wavelength of 485 nm and an emission wavelength of 520 nm. Orbital averaging was used at a radius of 3 mm.

### 2.9 Human serum

Peripheral blood was taken from healthy volunteers according to the University of Oxford local protocols and informed consent was obtained. Blood was left to clot at room temperature for 2 h and was subsequently centrifuged at 5000×*g* for 15 min at 4 °C. Serum was collected from the supernatant and used in experiments.

### 2.10 Animal tissue preparation

Male C57BL/6 J mice were terminally anaesthetized with Isoflurane, the trachea was exposed and the thorax was opened to expose the lungs. A 0.8 mm wide plastic tube was inserted into the trachea through an incision and attached to a 1 ml syringe. Lungs were washed three times with 0.7 ml PBS/10 mM EDTA by injection and withdrawal of solution. The peritoneum was lavaged with 10 ml ice cold PBS/2 mM EDTA and cells were collected for further use. Red blood cells were lysed with BD Pharm Lyse™ (BD Biosciences, Oxford, UK) as instructed by the manufacturer and were re-suspended in 0.5 ml PBS/1% FBS for further use.

### 2.11 Flow cytometry

Lung and peritoneal harvested cells were blocked with 10 μg/ml rat anti-mouse CD16/CD32 (AbD Serotec, Oxford, UK) and 1.17 mg/ml murine immunoglobulin G (IgG) (Jackson ImmunoResearch Laboratories, PA, USA) in Flow Cytometry buffer (PBS; 2% FBS, 25 mM HEPES, 5 mM EDTA) for 15 min on ice. Cells were stained with anti-CD45 (30-F11; BD Pharmigen), anti-CD11c (HL3; BD Pharmigen), anti-Siglec F (E50-2440; BD Pharmigen), anti-CD11b (M1/70; Biolegend, London, UK) and anti-F4/80 (CI:A3-1; Bio-rad) at 2 μg/ml in Flow Cytometry buffer for 30 min on ice protected from light. Cells were washed and pelleted at 5000×*g* for 10 min before being re-suspended in Flow Cytometry buffer. Samples were run on a Dako Cyan ADP flow cytometer (Beckman Coulter Ltd., High Wycombe, UK) and analyzed with FlowJo 10 software (Tree Star Inc, Ashland, USA). For cell counting, CountBright™ Absolute Counting Beads (Life Technologies) were used as instructed by manufacturer’s guidelines.

### 2.12 Quantitative Polymerase Chain Reaction (qPCR) (Table 1)

RNA extraction was carried out with the RNeasy® kit (Qiagen, Manchester, UK) and RNA quality was verified with a ND-100 spectrophotometer (Nano Drop Technologies, DE, USA). Complementary DNA (cDNA) was reverse transcribed from 400 to 600 ng RNA using the QuantiTect® Reverse Transcription kit (Qiagen) according to the manufacturer’s instructions. Twenty to thirty nanograms of cDNA were used as a template in qPCR experiments using specific primers (500 nM) and 2X Sybr® Select Master Mix as the detection chemistry (Life Technologies). The thermal profile included one phase at 95 °C for 5 min, second phase of 40 cycles at 95 °C for 20 s, 60 °C for 20 s and 72 °C for 20 s and last phase of 72 °C for 5 min. Melt curve analysis was run after every experiment. The experiments were carried out with a Step One Plus ™ platform (Applied Biosystems, MA, USA) and analyzed with the StepOne™ software. Cycle threshold (Ct) values were determined and relative mRNA contents were inferred from normalization of the gene of interest expression to that of the housekeeping gene (ΔCt). Relative expression results were plotted as (2^−^^ΔCt^).

### 2.13 Statistical analysis

All data are reported as mean + SEM of n = 3–5 independent experiments. Statistical analysis was carried out with GraphPad Prism 6.0 (CA, USA) using one- or two-way ANOVA with Dunnett’s or Sidak’s post hoc multiple comparisons tests, respectively. Non parametrical Kruskal–Wallis comparisons were carried out with Dunn’s post hoc multiple comparisons test, where data did not follow a normal distribution. Results were considered significant when p < 0.05.

## 3 Results

### 3.1 Cell density and phagocytic meal optimization

We first compared commercially available phagocytic meals to use in our *in vitro* assay. We cultured BMDMs (2.5 × 10^4^ cells per well) with an equal mix of 200 μg/ml pHrodo red *S. aureus* bioparticles and 200 μg/ml FITC-conjugated Zymosan A or FITC-conjugated latex beads for 1 h and took images using confocal microscopy. As shown in Fig.1D and E, pHrodo-labeled *S. aureus* bioparticles emitted red signal only when within macrophages. Unphagocytosed pHrodo *S. aureus* bioparticles gave no red signal (see yellow arrows in Fig.1D and E). In contrast, both FITC-conjugated Zymosan and latex beads were constitutively fluorescent and were observed following phagocytosis (see co-localization with *S. aureus* bioparticles in Fig.1D), but also when bound to macrophage membranes (white arrows) and free in the supernatant (orange arrows). For this reason, we decided to use the pHrodo-labeled bioparticles for the remainder of this study.

**Fig. 2:**
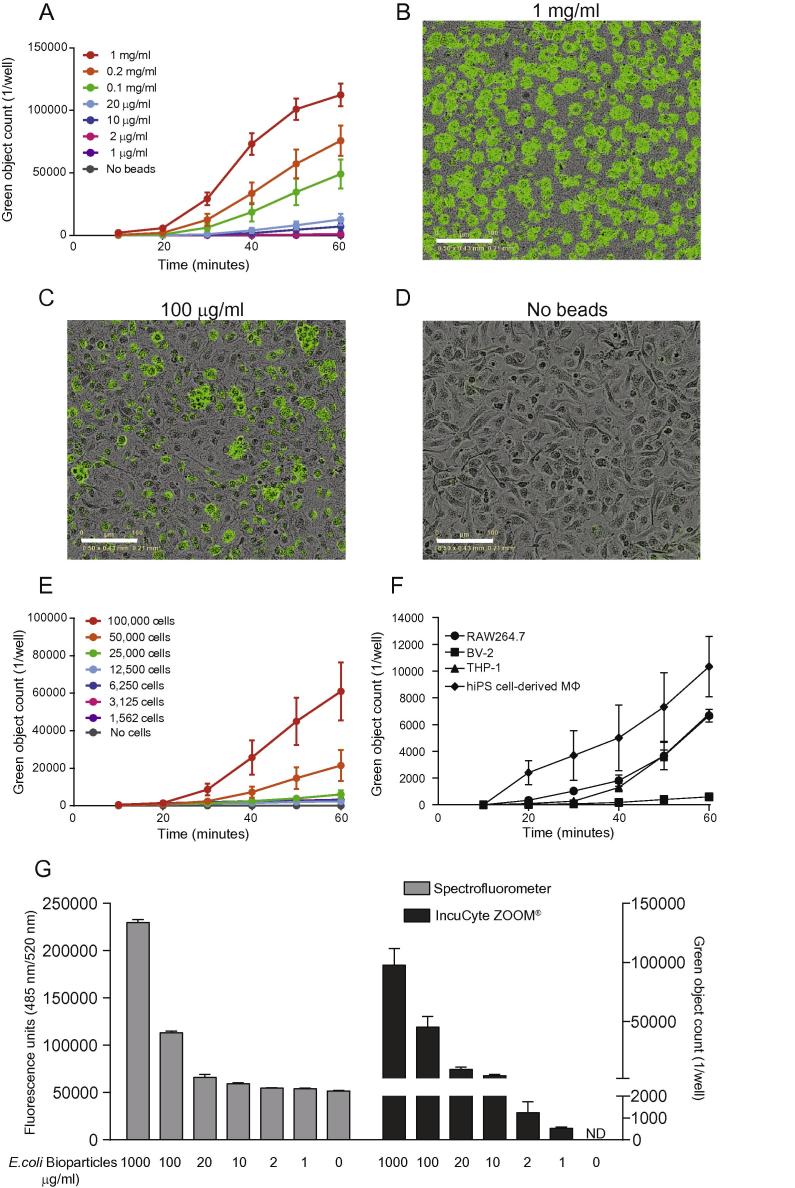
BMDM phagocytosis is pathogen particle number and cell density-dependent. (A) BMDMs (10^5^) from male C57BL/6J mice were incubated with green *E. coli* bioparticles (1 mg/ml to 1 μg/ml) and fluorescence emission was measured in the IncuCyte imaging platform every 10 min for one hour at 37 °C. (B)–(D) Representative images from one experiment at one hour. (E) BMDMs (100,000–1562 cells per well) were incubated with 200 μg/ml green *E. coli* bioparticles for one hour at 37 °C and fluorescence emission was measured in the IncuCyte imaging platform with 10 min intervals. (F) RAW264.7, BV-2, THP-1 and hiPS cell-derived macrophages (5 × 10^4^) were incubated with 200 μg/ml green *E. coli* bioparticles and fluorescence emission was measured in the IncuCyte imaging platform every 10 min for one hour at 37 °C. (G) Side-by-side comparison of green *E. coli* bioparticle phagocytosis by 100,000 BMDMs measured with a spectrofluorometer and the IncuCyte system. Data are presented as mean ± SEM from 3 to 4 biological replicates in technical duplicates, apart from F and G where data are from one experiment with two technical replicates and are presented as mean ± SD. Scale bar 100 μm.

We then sought to optimize the IncuCyte platform for pathogen particle number and cell density. Adherent BMDMs (1 × 10^5^ cells per well) were seeded for 2 h and fed with serially diluted *E. coli* bioparticles for 1 h. Fig.2A shows that phagocytosis was particle number-dependent with representative images shown in panels B–D. In addition, bioparticle uptake by different BMDM densities challenged with 200 μg/ml *E. coli* bioparticles followed a cell number-dependent pattern (Fig.2E). Based on these pilot experiments we decided to use a density of 50,000 BMDMs per well and concentration of 200 μg/ml *E. coli* bioparticles for the remainder of the study. A time lapse movie of 50,000 BMDMs phagocytosing 200 μg/ml *E. coli* bioparticles is shown in Supplementary Fig. 1. Finally, we measured the phagocytosis rate of several murine and human macrophage lines and hiPS cell-derived macrophages (Fig.2F). hiPS cell-derived macrophages showed a remarkably high uptake rate, whereas RAW264.7 cells and THP-1 exhibited similar *E. coli* bioparticle uptake rates. In contrast, BV-2 microglia cells phagocytosed fewer *E. coli* bioparticles.

A commonly used technique to measure phagocytosis of fluorescent particles is Spectrofluorometry. Comparison of the IncuCyte platform with a Spectrofluorometry-based system revealed a higher degree of phagocytosis-sensitivity with the IncuCyte platform (Fig.2G). *E. coli* bioparticle engulfment measured by Spectrofluorometry had a detection limit of 20 μg/ml bioparticles in comparison with the IncuCyte which could detect signal over background with as few as 1 μg/ml of particles.

Collectively, these results highlight the higher sensitivity and accuracy of the IncuCyte over Spectrofluorometry-based and confocal microscopy platforms for quantifying macrophage phagocytosis of pathogen bioparticles.

### 3.2 Measurement of inhibitor and opsonin activity

**Fig. 3:**
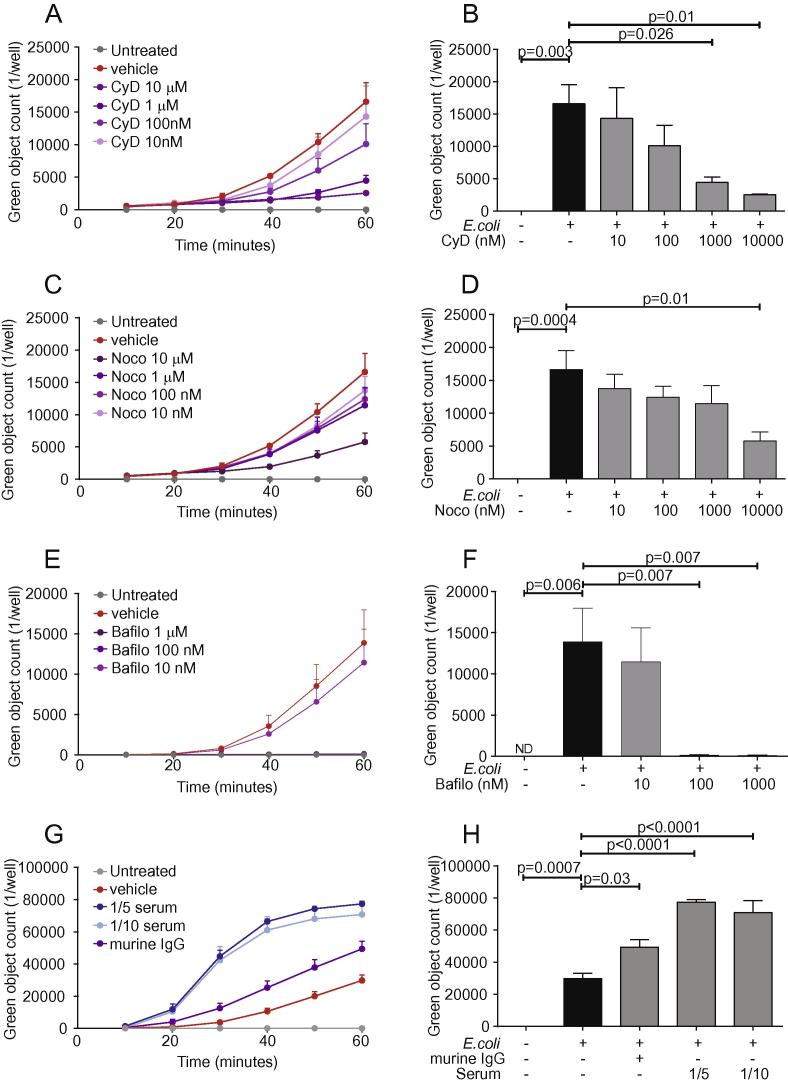
Macrophage phagocytosis is inhibited by actin and microtubule inhibitors and enhanced by opsonization. (A)–(D) BMDMs (5 × 10^4^) from C57BL/6J male mice were pre-treated with the actin and microtubule inhibitors Cytochalasin D and Nocodazole at the indicated concentration for one hour and were then incubated with 200 μg/ml green *E. coli* bioparticles at 37 °C for another hour. (E) and (F) BMDMs (5 × 10^4^) from C57BL/6J male mice were pre-treated with the vacuolar H^+^-ATPase inhibitor Bafilomycin A1 at the indicated concentration for one hour and were incubated with 200 μg/ml green *E. coli* bioparticles at 37 °C for another hour. (G) and (H) 200 μg/ml green *E. coli* bioparticles were opsonized with murine IgG or diluted human serum for 30 min at 37 °C and were then administered to 50,000 BMDMs from C57BL/6J male mice for one hour. (A), (C), (E) and (G) demonstrate the kinetics of phagocytosis at 10 min intervals. Fluorescence emission from all experiments was measured in the IncuCyte imaging platform and statistical analysis was carried out with one-way ANOVA with Dunnett’s multiple comparisons post hoc test. Data are presented as mean ± SEM from 3 to 5 biological replicates in technical duplicates.

Having optimized the IncuCyte phagocytosis assay, we wanted to further validate the quantitative imaging platform with known modulators of phagocytosis. We first pre-treated BMDMs with the actin inhibitor Cytochalasin D or the microtubule inhibitor Nocodazole for 1 h prior to the addition of *E. coli* bioparticles. Both Cytochalasin D and Nocodazole reduced the number of phagocytosed bioparticles in a concentration-dependent manner (Fig.3A and C). Nocodazole decreased *E. coli* bioparticle uptake by 65% at 10 μM (p = 0.01), whereas Cytochalasin D was a more potent inhibitor of phagocytosis (73% reduction at 1 μM, p = 0.026) (Fig.3B and D). In addition, pre-treatment of BMDMs for 1 h with Bafilomycin A1, an inhibitor of the phagolysosome V-ATPase, resulted in ablation of the fluorescent signal above the concentration of 100 nM (p = 0.007), demonstrating the key role of phagolysosome acidification for detection of pHrodo-labeled particles (Fig.3E and F).

To address whether enhancement of BMDM phagocytosis could also be quantified by the IncuCyte platform, we opsonized *E. coli* bioparticles with diluted human serum from healthy individuals or murine IgG and then delivered the meal to 50,000 BMDMs. Coating of the bioparticles with murine IgG and human serum significantly enhanced *E. coli* bioparticle phagocytosis (Fig.3G and H). Murine IgG increased phagocytosis 2-fold (p = 0.03), while human serum augmented particle ingestion by 2.4–2.6× (p < 0.0001). In addition, the phagocytosis curves of BMDMs treated with human serum-coated bioparticles exhibited a marked shift to the left, which indicates an increased rate of bioparticle uptake as confirmed by the slope metric (Human Serum 1/10 = 0.08, Human serum 1/5 = 0.08 and murine IgG = 0.04) (Fig.3G). A time lapse movie of human serum-pre-treated *E. coli* phagocytosis by BMDMs is shown in Supplementary Fig. 2.

Taken together, these results demonstrate that macrophage phagocytosis of bioparticles in real time is significantly reduced by actin polymerization and microtubule inhibitors and is augmented by opsonization of bioparticles. The results obtained with Bafilomycin A1 further supports the view that this phagocytosis methodology only detects uptake into the acidified phagolysosome. These findings suggest that, for the first time, there is a phagocytosis imaging platform that provides the opportunity to study the kinetics of novel chemical compounds or treatments that inhibit or enhance macrophage phagocytosis.

### 3.3 Differential pattern of phagocytic activity among different macrophage populations

One technical limitation of some macrophage phagocytosis assays is that they require large cell numbers in order to yield a detectable signal. We decided to test the ability of the IncuCyte platform to quantify pathogen phagocytosis by different resident macrophage populations, specifically, alveolar and resident peritoneal macrophages.

**Fig. 4:**
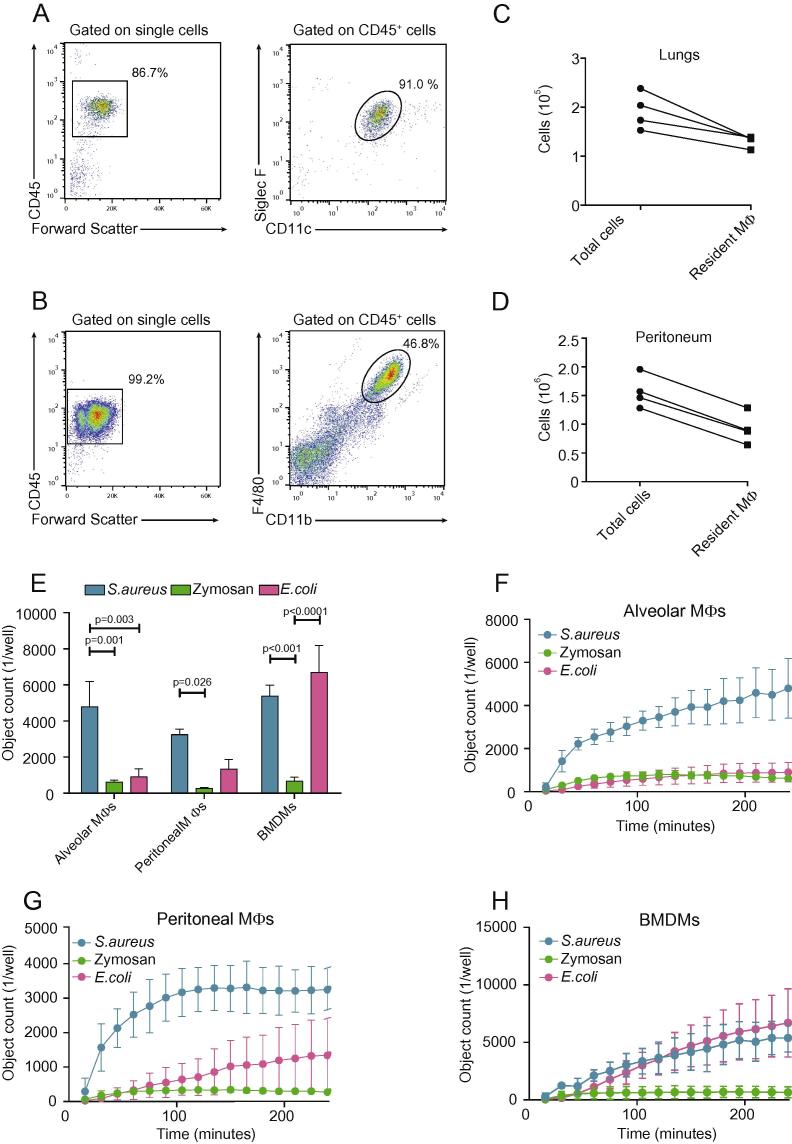
Primary murine macrophage populations exhibit differential efficiency of phagocytosis of different pathogens. C57BL/6J murine lungs and peritoneal cavities were lavaged and cells were lysed to remove red blood cells. (A) Alveolar macrophages were defined as Siglec F^+^ and CD11c^+^ cells within the CD45^+^ cell population of total lung cells. (B) Peritoneal resident macrophages were defined as F4/80^+^ and CD11b^+^ cells within the CD45^+^ cell population of total peritoneal cavity cells. (C) and (D) Macrophage numbers were inferred from total numbers and the Flow Cytometry gating strategy. (E) Alveolar, peritoneal and BMDMs (12,500 cells) were treated with 200 μg/ml green sterile Zymosan, *E. coli* and red *S. aureus* bioparticles for four hours and fluorescence emission was measured in the IncuCyte imaging platform every 15 min. (F)–(H) Kinetics of pathogen phagocytosis; 12,500 alveolar (F), peritoneal (G) and murine BMDMs (H) over four hours measured in the IncuCyte platform every 15 min. Data at four hours are presented as mean ± SEM from 4 biological replicates in technical duplicates. Statistical analysis was carried out with a two-way ANOVA and Tukey’s post hoc multiple comparisons test.

We harvested lung and peritoneal cells from naïve mice and stained them with antibodies to determine their number and macrophage purity (Fig.4A and B). Alveolar macrophages were identified as CD45^+^CD11c^+^Siglec F^+^ cells and peritoneal macrophages as CD45^+^F4/80^+^CD11b^+^ cells. The absolute numbers of these resident macrophage populations were generated from the total cell counts and the flow cytometry gating strategy shown in Fig.4C and D. We decided to use 12,500 alveolar macrophages, peritoneal macrophages or BMDMs per well and challenge them with 200 μg/ml *S. aureus*, Zymosan and *E. coli* bioparticles for 4 h with data collected every 15 min.

We hypothesized that each macrophage population would respond differently to selected non-opsonized pathogen bioparticles. Fig.4E shows that alveolar macrophages uptake *S. aureus* 8× more efficiently than Zymosan and 5× more efficiently than *E. coli* (p < 0.01) at 4 h, whereas BMDMs are effective in clearing both *S. aureus* (8×) and *E. coli* (10×) (p < 0.001 in comparison to Zymosan). Interestingly, resident peritoneal macrophages were not very efficient at pathogen bioparticle engulfment in comparison with other tissue-resident macrophages. Time kinetics showed that *S. aureus* is taken up more rapidly than *E. coli* and Zymosan by alveolar and peritoneal macrophages, whereas its uptake rate is similar to that of *E. coli* in BMDMs (Fig.4F–H).

The IncuCyte phagocytosis assay highlights key differences in the ability of different primary macrophage populations to phagocytose different pathogens. More importantly, the system allows users to monitor and accurately quantify phagocytosis by primary macrophage populations where only small numbers of cells can be obtained, which would not be feasible if using another phagocytosis methodology.

### 3.4 Long term phagocytosis assays with different macrophage seeding densities

**Fig. 5:**
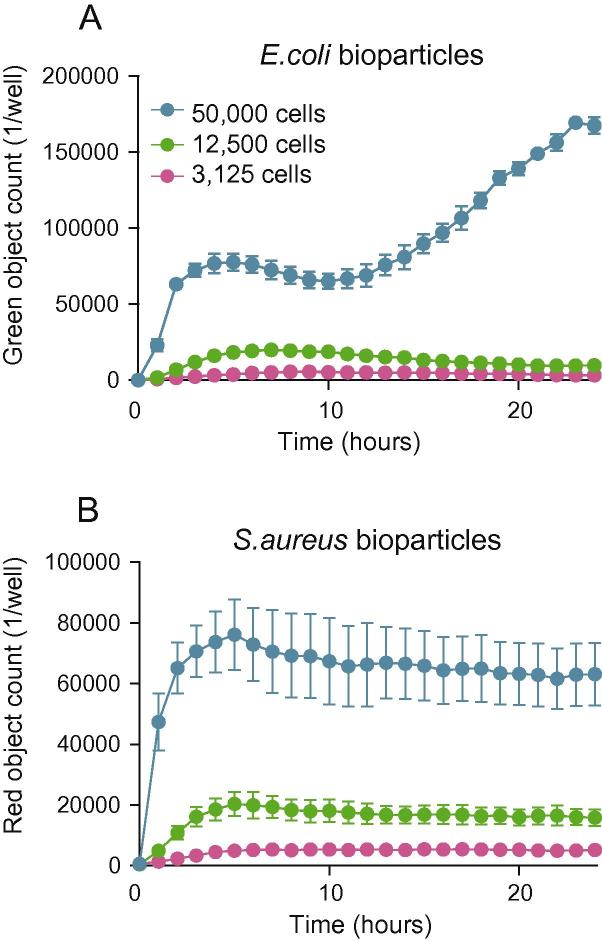
Long term *E. coli* phagocytosis is biphasic, whereas *S. aureus* uptake reaches a plateau at 4 h. (A) and (B) BMDMs from male C57BL/6J mice were seeded at 50,000, 12,500 and 3125 cells per well and incubated with 200 μg/ml green *E. coli* (A) or red *S. aureus* (B) bioparticles. Fluorescence signal was measured in the IncuCyte imaging platform every 20 min for 24 h at 37 °C. Data are presented as mean ± SEM from 3 biological replicates in technical duplicates.

To illustrate the IncuCyte platform’s ability to carry out long-term imaging and explore the full dynamic range of the assay, we seeded BMDMs at different densities (50,000–3125 cells per well) and performed a phagocytosis assay using 200 μg/ml green *E. coli* or red *S. aureus* bioparticles for 24 h (Fig. 5).

*E. coli* engulfment exhibited a biphasic response with peaks at 4 and 24 h, whereas *S. aureus* uptake reached a plateau at 5 h after which the signal remained constant until 24 h (Fig.5B). Taken together, these data further demonstrate the advantage of kinetic studies over single endpoint assays and exemplify the IncuCyte platform’s advantage over current methodologies to study macrophage phagocytosis in both short-term and long-term *in vitro* assays.

### 3.5 Macrophage polarization is an important determinant of phagocytic activity

**Fig. 6:**
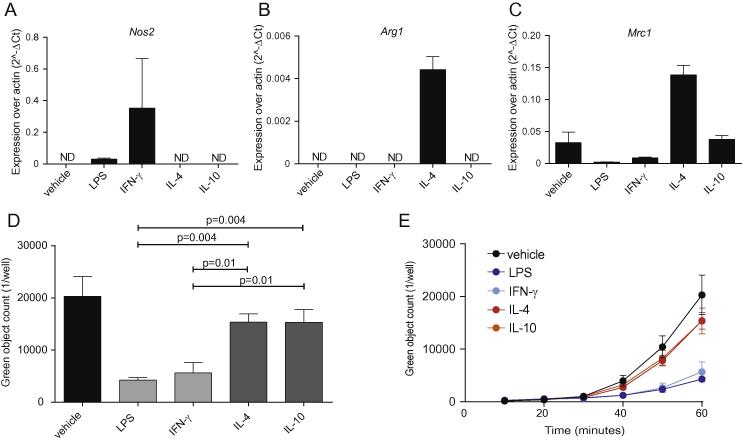
Macrophage polarization status affects phagocytic capacity. (A) BMDMs (2 × 10^5^) from C57BL/6J male mice were polarized by incubation with vehicle, 100 ng/ml LPS, 20 ng/ml IFN-γ, 20 ng/ml IL-4 or 10 ng/ml IL-10 at 37 °C for 16 h and (A) *Nos2*, (B) *Arg1* and (C) *Mrc1* mRNA expression was measured. (D) Polarized BMDMs (5 × 10^4^) from C57BL/6J male mice were subsequently incubated with 200 μg/ml green *E. coli* bioparticles for one hour. Fluorescence emission was measured in the IncuCyte imaging platform for one hour every 10 min. (E) Time kinetics of the macrophage polarization experiments. Data are presented as mean ± SEM from 4 biological replicates in technical duplicates. Statistical analysis was carried out with one-way ANOVA with Dunnett’s multiple comparisons post hoc test.

To explore how the IncuCyte system can be used to address scientific questions about macrophage biology, we polarized BMDMs to an M1 phenotype with 100 ng/ml LPS and 20 ng/ml IFN-γ or an M2 phenotype with 20 ng/ml IL-4 and 10 ng/ml IL-10 for 16 h and measured the mRNA expression levels of signature genes, such as *Nos2* for the M1 and *Arg1* and *Mrc1* for the M2 phenotype (Fig.6A–C).

Polarized BMDMs (5 × 10^4^) were incubated with *E. coli* bioparticles and phagocytosis was scored as before (Fig.6D and E). Fig.6D shows that treatment of BMDMs with LPS or IFN-γ led to significantly lower *E. coli* bioparticle uptake at 1 h (p < 0.01) compared to pre-treatment with IL-4 or IL-10. Moreover, M1 but not M2 polarization resulted in a significant reduction in phagocytosis compared to vehicle-pre-treated macrophages (p < 0.01).

These data show that polarization of primary macrophages using cytokines can modulate their capacity to engulf pathogens and that the M1 phenotype significantly impairs uptake of *E. coli* into acidified phagolysosomes. Therefore, questions relating to how primary macrophage polarization affects phagocytosis can be addressed with this system.

### 3.6 LPS exposure tolerizes macrophages and compromises phagocytosis

**Fig. 7:**
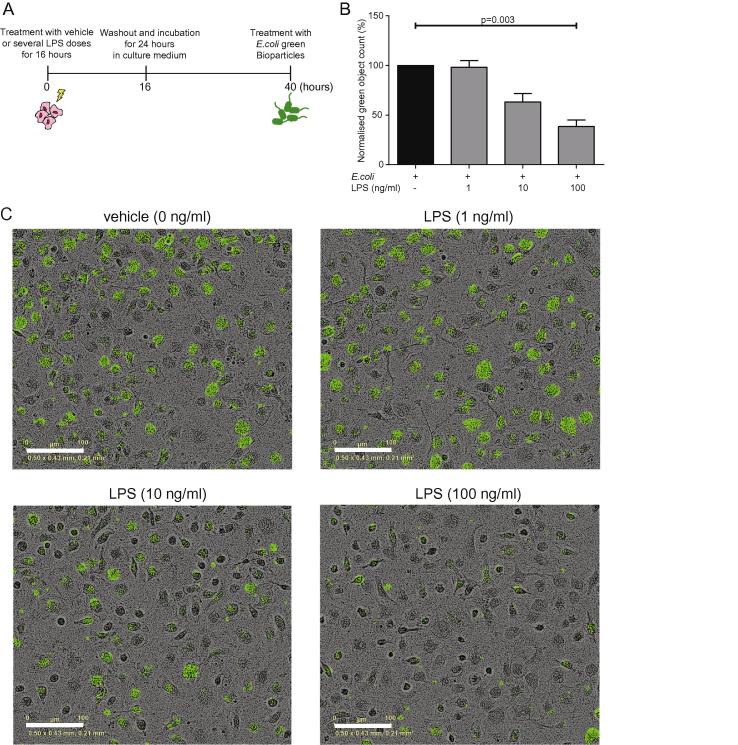
Pre-treatment with LPS leads to reduced *E. coli* phagocytosis by macrophages. (A) and (B) BMDMs (5 × 10^4^) were primed with a range of LPS doses for 16 h and cells were incubated for another 24 h at 37 °C in culture media, after which 200 μg/ml green *E. coli* bioparticles were incubated with the macrophages in the IncuCyte platform for another hour. Green object counts in vehicle-treated macrophages were set to 100% and object counts in LPS pre-treated macrophage groups within the same experiment were normalized to demonstrate% reduction compared to the control. (C) Representative images from one experiment at one hour. Data from 5 biological replicates in technical duplicates are presented as mean ± SEM. Statistical analysis was carried out with Kruskal-Wallis and Dunn’s multiple comparisons post hoc test. Scale bar is 100 μm.

The fact that M1 polarized BMDMs displayed weaker phagocytic activity in comparison with M2 macrophages prompted us to look into this observation in more detail. We therefore pre-treated BMDMs with a range of LPS concentrations for 16 h, washed and cultured the cells in macrophage culture media for a further 24 h (in the absence of LPS) and finally administered *E. coli* bioparticles for 1 h (Fig.7A). We hypothesized that LPS-pre-treated BMDMs would be tolerized and therefore respond differently to a subsequent presentation of *E. coli* bioparticles compared to vehicle-treated cells, resulting in altered kinetics of phagocytosis.

Pre-treatment of BMDMs with 100 ng/ml LPS resulted in reduced mRNA expression levels of cytokines and chemokines, such as *Tnf*, *Ccl2* and *Ccl3*, and increased the expression of *Il6*, *Nos2* and *Ccl5* in comparison with untreated macrophages (data not shown). We found that LPS pre-treatment inhibited BMDM phagocytosis in a concentration-dependent manner (Fig.7B). Pre-treatment with 100 ng/ml LPS resulted in 62% reduction in phagocytosis when compared to vehicle-treated BMDMs (p = 0.003, representative images from one experiment are shown in Fig.7C). Taken together, these results imply that one possible reason why M1 polarized macrophages are less efficient at *E. coli* bioparticle engulfment is TLR stimulus-mediated tolerance.

## 4 Discussion

In this study we describe a novel imaging platform to quantify macrophage phagocytosis *in vitro*. This technology combines pH-dependent dye particle labeling, the acquisition of images in real time and operator-independent image analysis. We have exemplified the quantitative aspect of this technology platform using drugs that inhibit cytoskeletal changes and lysosome acidification. Furthermore, we show the ability to quantify enhancement of phagocytosis in primary macrophages using repeated measures every 10 min. We hereby show that this technology can be successfully applied to overcome common technical limitations when carrying out phagocytosis assays *in vitro*.

Foreign object phagocytosis has traditionally been studied as the uptake of microspheres, fluorescent pathogens or sheep erythrocytes. The advantage of using the commercially available pHrodo pathogen bioparticles is that they only fluoresce when they are localized in an acidic micro-environment, like that of the phagolysosome. Our data clearly demonstrate that pHrodo-labeled bioparticles do not fluoresce when bound to the macrophage membrane or when free in the culture supernatant, in contrast to latex beads or FITC-labeled pathogens. Microscopy [26], [27] and flow cytometry techniques [28] have been used to visualize and quantify phagocytosis of *E. coli* bioparticles by non-adherent phagocytic cells or macrophage cell lines. However, primary macrophages can be stressed when detached from plastic to be run on a flow or imaging flow cytometer and quantification of phagocytosis from microscopy image analysis is of low throughput and potentially open to operator bias. The platform we present here uses microscopy to capture and quantify primary murine macrophage phagocytosis and has no negative impact on cell viability.

An innovative technical feature of the IncuCyte is the data analysis software. The operator is required to set up definition processes which are then applied universally to all images of interest eliminating the potential for bias. Data acquisition stringency can be increased via modifications in the cell size and fluorescence intensity threshold and closely localized objects can reliably be distinguished from each other using the edge split software function.

To investigate the sensitivity of the IncuCyte platform, we titrated the particle numbers in the phagocytic meal given to primary murine macrophages. Initial experiments showed that the IncuCyte could reliably detect fluorescent signal from 20× fewer pathogen particles than the spectrofluorometer. To further illustrate the power of this novel platform to effectively quantify particle fluorescence from limited primary macrophage sources, we isolated murine alveolar and peritoneal macrophages and compared them side by side with BMDMs (12,500 cells per well).

Foreign particle engagement with scavenger, Fcγ or complement receptors on the macrophage plasma membrane leads to the activation of small GTPases of the Rho family and the induction of actin filament re-arrangement and microtubule formation [29], [30], [31], [32], [33]. Pharmacological inhibition with the actin re-arrangement inhibitor Cytochalasin D and the microtubule inhibitor Nocodazole highlighted the importance of these signaling pathways in phagosome formation and transport within the macrophage, as both drugs abrogated *E. coli* uptake by macrophages. The observed differences in the efficiency of the two drugs suggest that the IncuCyte platform might prove a valuable tool in deciphering the contribution of individual proteins or regulatory complexes in the phagocytic process. In addition, pre-treatment of macrophages with the inhibitor of the phagolysosome V-ATPase Bafilomycin A1, ablated signal detection. The V-ATPase is a protein complex located on the phagolysosome membrane and is the major mechanism responsible for the inhibition of phagolysosome passive H^+^ leak into the cytosol [34]. Our data confirm that the signal quantified by our assay is pH sensitive and therefore corresponds to the late phagosome stage of *E. coli* phagocytosis when bioparticles have been completely engulfed by the macrophages.

Phagocytes are equipped with FcγRs and express receptors for C1 peptide and C3 fragments, such as C3b and iC3b that mediate the opsonization of coated cells, microbes or surfaces [35], [36], [37], [38]. Moreover, immunoglobulins have been shown to increase the early steps of phagocytosis in a concentration-dependent manner [39] and opsonization accelerates the clearance of microbes, such as fungi [40], [41], viruses [42] and parasites [43]. Our experiments demonstrate that opsonization of the phagocytic meal with human serum or murine IgG increases the ability of macrophages to clear *E. coli*. Although it was originally shown that there are differences in the mechanisms and downstream signaling between the two processes [44], [45], [46], the fact that complement receptors have been reported to act independently of complement and in an antibody-mediated manner raises the possibility that it may act in synergy with immunoglobulins to clear serum-coated *E. coli* particles more potently than murine IgG-coated particles [47], [48], [49]. Furthermore, serum contains a plethora of other opsonins, such as collectins, fibronectin and pentraxins [50], which can have an additive effect on complement’s functions and may explain the significantly higher serum coated *E. coli* uptake by BMDMs compared to murine IgG in our assays.

It has been shown that the functions of different tissue-resident macrophages are largely shaped by their micro-environment [51], [52] and macrophages are integrated with their niche to perform tissue-specific functions in homeostasis [53]. We therefore hypothesized that macrophage populations from different anatomical sites would exhibit differences in phagocytic activity for different pathogens. To the best of our knowledge, we are the first to directly compare phagocytosis between different primary macrophage populations and show significant variation in macrophage pathogen preference over a range of phagocytic meals. Alveolar macrophages displayed preference for *S. aureus*, in contrast to BMDMs which cleared *E. coli* and *S. aureus* with equal efficiency.

One explanation could be the differential expression of scavenger receptors, C-type lectins and integrins between macrophage populations, such as alveolar, peritoneal and microglia cells [54]. This differential pathogen uptake by macrophages is also illustrated in our long-term assays, where the same macrophage population engulfed *E. coli* bioparticles with different kinetics from *S. aureus* biparticles. These data highlight the ability of the IncuCyte platform to carry out long-term *in vitro* assays and will facilitate the study of macrophage responses to infection with different pathogens.

Macrophages are primed by a wide range of PAMPs and DAMPs and acquire different phenotypes according to the stimulus that activated them [55]. M1 macrophages secrete pro-inflammatory cytokines and chemokines, while M2 macrophages specialize in wound healing and resolution of inflammation [56], [57]. The potential of the IncuCyte to be used as a tool in macrophage biology studies is exemplified by our finding that M2 macrophages displayed a significantly higher phagocytic capacity than M1 macrophages. Our results agree with Li et al. who showed that M2 macrophages exhibited a higher uptake of *H. ducreyi* and *E. coli* than M1 macrophages [58]. It was recently reported that macrophage colony stimulating factor-differentiated macrophages are more efficient at phagocytosis of rituximab-opsonized B-CLL tumor cells than granulocyte-macrophage colony stimulating factor-differentiated macrophages [59]. In contrast to Varin et al., we did not observe an IL-4-driven inhibition of pathogen phagocytosis which might be related to the different bacterial pathogens used in the two studies [60].

Excessive toll-like receptor (TLR) activation of macrophages can lead to infection, chronic inflammation and autoimmunity. Several mechanisms of TLR activation regulation have been described so far, including signaling and transmembrane inhibitors, soluble decoy TLRs and reduction in the expression of TLR genes [61]. Another mechanism by which macrophage activation is restrained is tolerance induction. Specifically, endotoxin tolerance is defined as a hyporesponsive state induced in immune cells when repeatedly challenged with low doses of endotoxin [62]. Endotoxin tolerant macrophages bear some similarities with the M2 phenotype and decrease the expression of pro-inflammatory cytokines and chemokines [63]. In contrast, they promote secretion of anti-inflammatory cytokines and expression of scavenger receptors [62], [63]. Furthermore, genes that encode tissue damage products are epigenetically silenced, while others expressing anti-microbial effectors are induced in tolerant macrophages [64].

Here we studied the effect of endotoxin tolerance on *E. coli* phagocytosis after pre-treatment of macrophages with purified LPS as a possible explanation for the decreased phagocytic capacity in M1 macrophages. Our observation that *E. coli* phagocytosis was inhibited in a concentration-dependent manner suggests that tolerance to M1 stimuli might be an important cause of impaired phagocytic capacity of macrophage populations *in vivo*. Despite this, our data are in seeming disagreement with a previous report, where human LPS tolerant monocytes displayed enhanced phagocytosis of *E. coli* bacteria [65]. Similarly, Pena et al. showed that peripheral blood mononuclear cells tolerized with LPS overexpressed macrophage receptor with collagenous structure (MARCO) and CD23 [66]. The observed discrepancy between our current report and others might be caused by interspecies differences. Future work is therefore needed to understand why endotoxin tolerance leads to suppressed phagocytosis of *E. coli* particles by murine BMDMs. The availability of a quantitative macrophage phagocytosis assay will allow the molecular basis of LPS tolerance effects to be explored in more detail.

Like all phagocytosis assays the IncuCyte-pHrodo platform comes with some limitations. It is limited by the nature and range of commercially available and phagolysosome-restricted phagocytic meals. The use of killed pathogens does not reflect the situation *in vivo* where highly dividing and virulent strains of pathogens interact with and evade the host’s immune system. In addition, the platform detects fluorescence in one green and one red channel each with a narrow emission spectrum that restricts the user from additional labeling of cell compartments (Table 2).

In conclusion, we consider that the IncuCyte real time imaging platform represents an important improvement that can be used as a complement to current methods to study macrophage phagocytosis *in vitro*. Its main advantage is real time acquisition of kinetic data in short and long-term assays that allows quantitative data analysis via its user-friendly software package. Because of its high sensitivity, this system outperforms many current techniques used to quantify phagocytosis and allows the study of primary murine macrophage populations where cell numbers are a limiting factor. This novel platform will allow us to address complex biological questions relating to human and murine macrophage phagocytosis and to screen for positive and negative modulators of this fundamental cellular process.

## Conflict of interest

The authors declare that they have no conflicts of interest.

## Figures and Tables

**Table 1:** Primers used for detection of M1 and M2 polarization markers.

Gene	Primer	Primer sequence (5′ → 3′)
*Mm_Nos2*	Sense	CAAGCCCTCACCTACTTCCTG
	Antisense	AATCTCTGCCTATCCGTCTC

*Mm_Arg1*	Sense	ACCTTGGCTTGCTTCGGA
	Antisense	CTGTCTGCTTTGCTGTGAT

*Mm_Mrc1*	Sense	TGTGTAGTTGTGATTGGTGG
	Antisense	TGGAGTAGTGGTTGGAGAAA

*Mm_Actg1*	Sense	CCAACAGCAGACTTCCAGGATT
	Antisense	CTGGCAAGAAGGAGTGGTAACTG

**Table 2:** Comparison of the IncuCyte ZOOM® platform with other commonly used techniques for phagocytosis quantification.

	IncuCyte ZOOM®	Spectrofluorometry	Confocal microscopy	Flow cytometry	Imaging flow cytometry
Sensitivity	+++	++	+++	++	++
Magnification	++	N/A	+++	N/A	+++
Cell viability	+++	+++	+++	+	+
Real time kinetics	+++	+++	N/A	N/A	N/A
Low cell numbers	+++	+	+++	++	+++
Potential for operator bias	+	+	+++	+	+

## Appendix A Supplementary data

Supplementary video 1 *E. coli* bioparticles are engulfed by murine BMDMs. Time lapse movie of murine 50,000 BMDMs phagocytosing 200 μg/ml green *E. coli* bioparticles. Images were taken for one hour every 4 min. Movie speed is 3 frames/second.

Supplementary video 2 *E. coli* bioparticle phagocytosis by murine BMDMs is enhanced by coating with human serum. Time lapse movie of murine 50,000 BMDMs phagocytosing 200 μg/ml green *E. coli* bioparticles pre-treated with human serum for 30 min at 37 °C. Images were taken for one hour every 4 min. Movie speed is 3 frames/second.
