# Supplementary figures and images for: A novel real time imaging platform to quantify macrophage phagocytosis
Source: Biochem Pharmacol. 2016 Sep 15;116:107–19. doi: 10.1016/j.bcp.2016.07.011 (PMC5012892; doi:10.1016/j.bcp.2016.07.011)

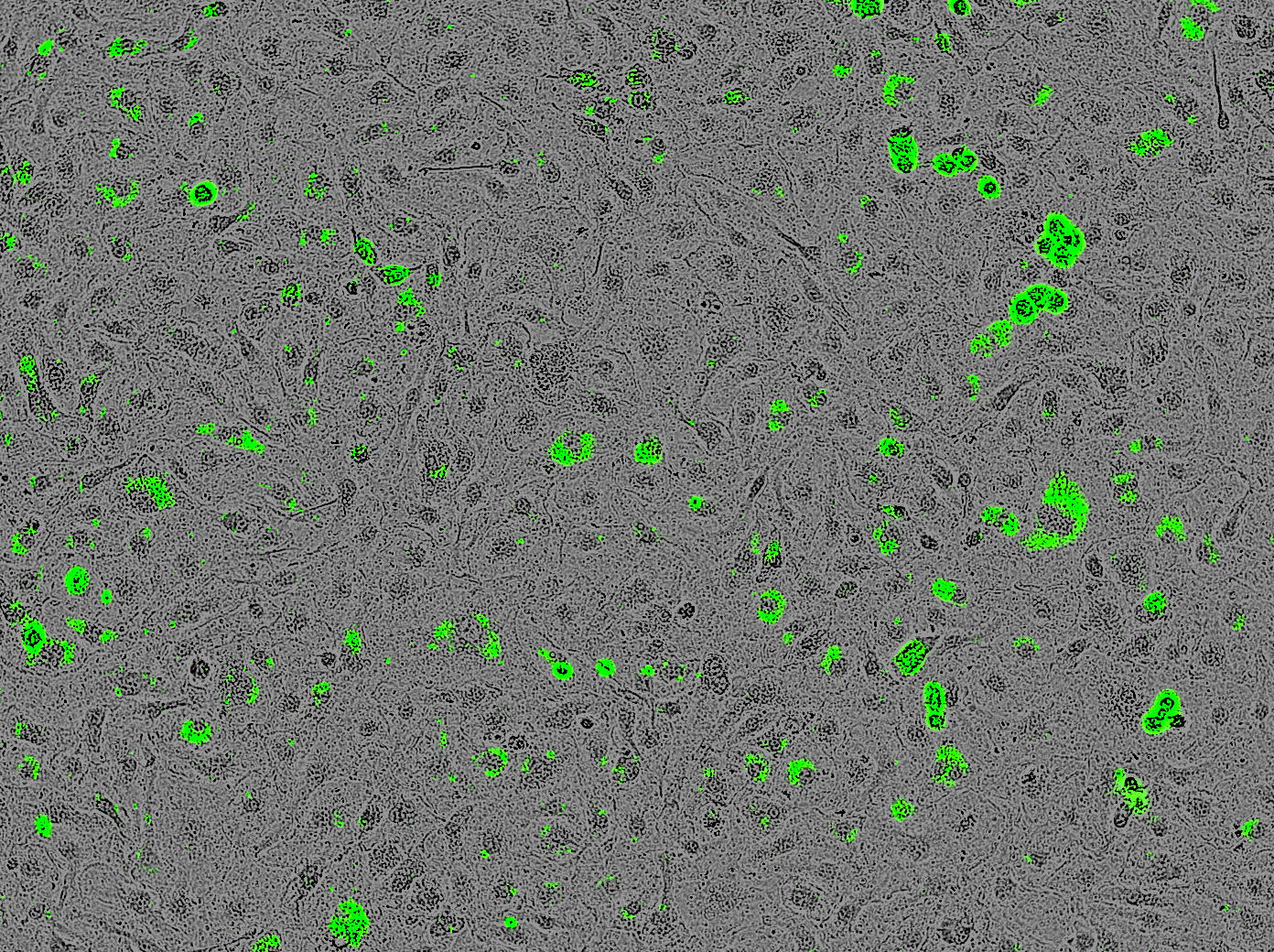

Supplement: Supplementary video 1 — E. coli bioparticles are engulfed by murine BMDMs. Time lapse movie of murine 50,000 BMDMs phagocytosing 200 μg/ml green E. coli bioparticles. Images were taken for one hour every 4 min. Movie speed is 3 frames/second. [file mmc1.jpg]

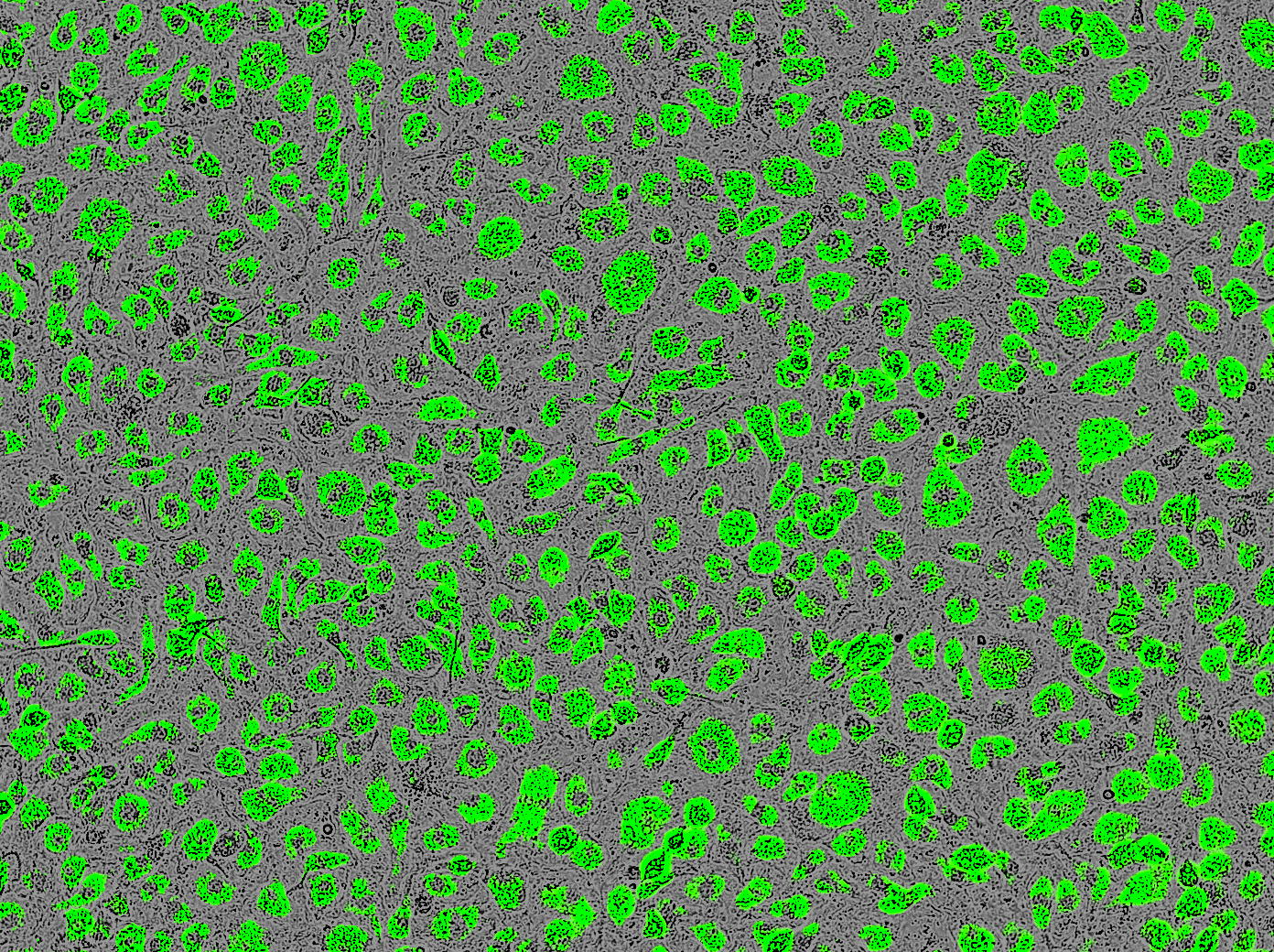

Supplement: Supplementary video 2 — E. coli bioparticle phagocytosis by murine BMDMs is enhanced by coating with human serum. Time lapse movie of murine 50,000 BMDMs phagocytosing 200 μg/ml green E. coli bioparticles pre-treated with human serum for 30 min at 37 °C. Images were taken for one hour every 4 min. Movie speed is 3 frames/second. [file mmc2.jpg]
